# Participation in disease management programs and major adverse cardiac events in patients after acute myocardial infarction: a longitudinal study based on registry data

**DOI:** 10.1186/s12872-020-01832-3

**Published:** 2021-01-06

**Authors:** Christian Fischer, Jens Höpner, Saskia Hartwig, Michel Noutsias, Rafael Mikolajczyk

**Affiliations:** 1 Institute of Medical Epidemiology, Biometrics and Informatics, Medical Faculty of Martin Luther University Halle-Wittenberg, Magdeburger Straße 8, 06112 Halle (Saale), Germany; 2 Mid-German Heart Center, Department of Internal Medicine III (KIM III), Division of Cardiology, Angiology and Intensive Medical Care, University Hospital Halle, Martin Luther University Halle-Wittenberg, Ernst-Grube-Str. 40, 06097 Halle (Saale), Germany

**Keywords:** Myocardial infarction, Heart attack, DMP, Rehabilitation, MACE, Outpatient, Health care, Coronary heart disease, Secondary prevention

## Abstract

**Background:**

Cardiovascular diseases are still the main cause of death in the western world. However, diminishing mortality rates of acute myocardial infarction (AMI) are motivating the need to investigate the process of secondary prevention after AMI. Besides cardiac rehabilitation, disease management programs (DMPs) are an important component of outpatient care after AMI in Germany. This study aims to analyze outcomes after AMI among those who participated in DMPs and cardiac rehabilitation (CR) in a region with overall increased cardiovascular morbidity and mortality.

**Methods:**

Based on data from a regional myocardial infarction registry and a 2-year follow-up period, we assessed the occurrence of major adverse cardiac events (MACE) in relation to participation in CR and DMP, risk factors for complications and individual healths well as lifestyle characteristics. Multivariable Cox regression was performed to compare survival time between participants and non-participants until an adverse event occurred.

**Results:**

Of 1094 observed patients post-AMI, 272 were enrolled in a DMP. An association between DMP participation and lower hazard rates for MACE compared to non-enrollees could not be proven in the crude model (hazard ratio = 0.93; 95% confidence interval = 0.65–1.33). When adjusted for possible confounding variables, these results remained virtually unchanged (1.03; 0.72–1.48). Furthermore, smokers and obese patients showed a distinctly lower chance of DMP enrollment. In contrast, those who participated in CR showed a lower risk for MACE in crude (0.52; 0.41–0.65) and adjusted analysis (0.56; 0.44–0.71).

**Conclusions:**

Participation in DMP was not associated with a lower risk of MACE, but participation in CR showed beneficial effects. Adjustment only slightly changed effect estimates in both cases, but it is still important to consider potential effects of additional confounding variables.

## Background

Cardiovascular diseases are still the main cause of death in the western world. In 2017, 37% of all deaths in Germany were caused by diseases directly affecting the cardiovascular system [1]. The two most frequent death-related diagnoses were coronary heart disease (CHD) and acute myocardial infarction (AMI) [2]. Due to standardized acute revascularization management by interventional cardiology, AMI mortality rate has been decreasing in the last three decades [3]. However, AMI survivors are a high-risk group for death or another severe health event, especially if they are affected by additional risk factors such as smoking, obesity or diabetes [4]. To avoid subsequent severe health events, secondary prevention is needed [5].

Cardiac rehabilitation (CR) is one of the best-known and most often recommended secondary prevention approach after AMI [5–9]. However, in light of its high costs, delayed return home (in the case of in-patient treatment), and only a short period of intervention, the search for alternatives is well justified [10–13]. One such alternative is a disease management program (DMP) which has been introduced in Germany in 2002 to improve outpatient medical treatment quality and reduce costs in the health care system [14]. DMPs are structured treatment programs, coordinated by the patient’s general practitioner (GP). Since 2012, all statutory health insurance companies in Germany are required to offer DMPs based on the guidelines of the Federal Joint Committee to achieve nationwide homogeneity. Participating in a DMP is voluntary for the patients, and recommendation to a DMP is voluntary for the GPs. For patients, inclusion criteria are defined for enrollment in a DMP [15]. Patients, who meet the inclusion criteria for a specific DMP are asked by their GP or Health Insurance Company to enroll into DMP. For patients enrolled in the DMP coronary heart disease the program includes systematic control of medication, recommendations on nutrition and physical activity as well as advice on smoking cessation if relevant.

However, 18 years after the introduction of the first DMP for coronary heart disease, there is still insufficient evidence regarding the effectiveness of DMPs. Aside from the mandatory evaluations of insurance companies, only a few studies were published, and they mostly focused on DMPs related to diabetes mellitus [16–25]. Since DMPs were introduced all over Germany as a mandatory service, only observational studies can contribute further evidence on their performance [26].

In the federal state of Saxony-Anhalt, there were 75 deaths per 100,000 persons with AMI as recorded cause of death in 2016, which is 38.5% above the German average (55 deaths per 100,000 persons) [2]. In order to investigate the causes of this increased level, a regional registry of myocardial infarction in urban and rural regions of the federal state (RHESA) was established [27]. Patients who agreed to participate in RHESA are followed over time using questionnaires and health status information [28]. As one explanation of the elevated mortality could be suboptimal secondary prevention, we performed a follow-up of the RHESA participants that focused on secondary prevention programs.

Our aim was to assess the participation of AMI survivors in the secondary prevention programs and the association between the participation in secondary prevention and major cardiac outcomes including another AMI, stroke, percutaneous coronary intervention (PCI), coronary artery bypass graft (CABG) or death.

### Methods

#### Study design, study location, period of recruitment and participants

**Fig. 1:**
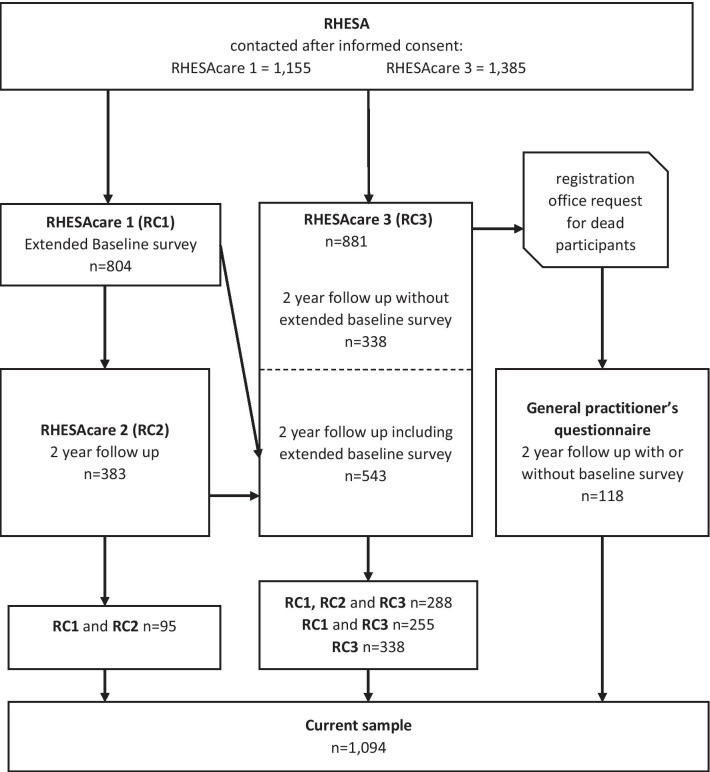
Data origin flow chart

We used data from follow-up of patients registered in RHESA. RHESA and its follow-up modalities were described elsewhere [27, 28]. In brief, RHESA was established to investigate the causes of the increased level of morbidity and mortality of AMI of the federal state Saxony-Anhalt [27] and collected information about all fatal and non-fatal myocardial infarctions in the city of Halle and in the rural region of the Altmark. In addition to collecting anonymous data, patients with AMI were asked during their hospital stay if they are willing to contribute their data and answer questionnaires in the future. Study instruments included hospital questionnaires filled out by medical staff, death certificates of the regionally responsible health office and documentations of emergency aid [28]. The recruitment for RHESA and its baseline information was obtained via questionnaires from physicians or study nurses in the respective hospitals since June 2013 and is ongoing. Between 2013 and 2017, a first follow-up named RHESA-Care1 (RC1) was conducted 6 weeks after hospital discharge (n = 804 patients participated); between 2015 and 2018, a second follow-up [RHESA-Care 2 (RC2)] was conducted, in which patients were contacted 2 years after their hospital discharge (n = 383) (Fig. 1). For the purpose of the current study, we conducted a third follow-up [RHESA-Care 3 (RC3)] of all patients from March to June 2019. This third follow-up focused on information about the occurrence of cardiac events: AMI, stroke, death, PCI or CABG, in addition to information regarding participation in DMPs or CR, co-morbidities, and socioeconomic factors as well as smoking/smoking cessations. In order to clarify if patients who died before the third follow-up had participated in DMPs, we contacted the doctors who signed their death certificates with a short questionnaire regarding their assessment of the cause of death and DMP participation. For the current study we included all participants, who took part in either RC1 and RC2 or RC3 (Fig. 1).

#### Exposure and covariates

In the current study we compared the long-term survival and cardiac health of AMI patients in terms of their participation in secondary prevention programs. We furthermore used the variables of obesity (≥ 30 kg/m^2^ vs. < 30 kg/m^2^ at the time of the initial AMI), hypertension (elevated arterial blood pressure diagnosed before or at the time of the initial AMI), and ST-elevated myocardial infarction (STEMI) versus non-ST-elevated myocardial infarction (NSTEMI) based on baseline questionnaire of RHESA as covariates in the cox regression models. To examine the factors that influence the participation in secondary prevention, we also used baseline information from the hospital questionnaires for each patient’s history with on smoking, diabetes, STEMI/NSTEMI, sex, obesity hypertension and age at the initial AMI as independent variables. Since analysis of age as a categorical variable did not indicate any nonlinearity, age was modelled as a continuous variable.

### Ethical approval

All participants provided written informed consent for participation in RHESA. The Ethics Committee of the Medical Faculty of the Martin-Luther University Halle-Wittenberg approved the initial study and the follow-up questionnaires.

### End points

The primary endpoint was survival time, starting 15 days after the initial AMI, until the occurrence of the first major adverse cardiac event (MACE) with a maximum of 24 months of observation time. In the composite endpoint, we included: AMI, stroke, PCI, CABG, and death (all-cause mortality and cardiac death were analyzed separately). As 2 weeks after AMI is the usual period for starting a subsequent cardiac rehabilitation according to Volume V of the German Social Code (SGB V) and to avoid immortal time bias [32], only those events which occurred at least fifteen days after the initial AMI were included in the study. In case of multiple outcomes, only the first was considered. Occurrence for all MACE as well as the beginning of participation in DMPs were self-reported by the patient or reported by the patient’s GP with an exact date. However, due to many participants only reporting the month, every event was recorded to have happened on the 15^th^ of the respective month. In the case of discordant information in different follow-up questionnaires, we used the information which was collected most closely following the reported event. Since enrollment into a DMP can occur at various times after the AMI, we considered DMP as a time-dependent exposure.

A known risk of using composite endpoints are dilution effects [29]. Thus, we defined two subtypes of MACE: MACE1 included only AMI, stroke, and death. MACE2 included AMI, stroke and death in addition to PCI and CABG. In the analyses of determinants for participation in secondary prevention, the endpoint was participation in a DMP or CR, respectively.

### Statistical analysis

Descriptive characteristics of the sample were reported as percentages and mean values with 95% confidence intervals (CI).

In order to identify determinants of DMP and CR, we obtained multivariable relative risks from a Poisson model with robust error variance as alternative to a logistic regression analysis for frequent outcomes [30]. In these models, participation in either DMP or CR was considered the main exposure while the outcomes were the ones specified above. We adjusted the model for all included covariates (smoking, diabetes mellitus, STEMI, sex, obesity, hypertension, and age). Since investigated covariates were potentially correlated, we tested for their multicollinearity, but no covariates needed to be excluded [31].

We analyzed the effects of CR or DMP participation on the occurrence of MACE using Cox proportional hazard regression models. We assessed assumption of proportional hazards for the Cox model by inspecting respective Kaplan–Meier plots (Additional file 1: Fig. 1).

Covariates for adjustment were selected based on the literature and directed acyclic graphs theory. They were: diabetes mellitus (diagnosed before or at the time of the initial AMI), smoking status (being smoker at the time of the initial AMI), sex, obesity, and age at the time of initial AMI. The models for CR and DMP participation were also mutually adjusted for these variables. Adjusting our analysis by socioeconomic status was not applicable, because of missing data, especially due to the GP’s questionnaire not including the corresponding items.

Some patients participated in DMP before their AMI—this time was censored to create a proper “time zero” of the time-dependent covariate [33]. In a sensitivity analysis, those who started participating in a DMP before AMI were excluded from the analysis.

Since the sample size was predefined, we investigated how strong the observed effects have to be, to provide statistically significant results. We estimated that a risk reduction of 18% or more could be detected at the significance level of *p* < 0.05 with 80% power. A risk reduction of 18% would be considered clinically relevant.

Besides social characteristics, patients taking part in either DMPs or CR could have a stronger motivation to change their lifestyle than those, who do not choose to participate. In such way, the participants of secondary prevention programs could be those with stronger motivation and likely better outcomes. This internal motivation is difficult to study. In an earlier analysis [44], we found that patients who stopped smoking after AMI (before CR) also had a higher probability of attending CR. Smoking cessation and attending CR were both possibly resulting from a higher internal motivation, which might also reduce MACE independently of CR. We compared effects of CR on MACE in those, who stopped smoking before CR and those who stopped later or did not stop.

All statistical analyzes were performed with SAS 9.4.

### Results

#### Sample characteristics

Of the 1385 participants who provided informed consent for the follow-up and were alive in May 2019, 881 (63.6%) participated in the third follow-up of RHESA and filled out the corresponding questionnaire after up to two reminders. Additionally, there were 95 people who participated in the follow-up 2 years after AMI but did not participate in the third follow-up. Still, because they provided the relevant information for the current analysis in the earlier questionnaire they were also included in the analysis. Furthermore, we received information about 118 patients who died before their second follow-up through a questionnaire filled out by their respective GPs. The median duration of follow-up for all patients was 24 months. For patients, who experienced an event, the median duration of follow-up was 8 months.

Of the 1,094 participants that were included in our study, about one quarter (24.9%) had been enrolled in a DMP while 58.5% took part in CR after treatment of the initial AMI, 189 patients (17.3%) participated both in DMPs and CR. Of all patients, 33.9% did not participate in either CR or a DMP (Table 1). CR participants were more likely to be also enrolled in DMP and vice versa. Of all DMP participants, 18.3% were enrolled in a DMP before the registered AMI. The remaining participants enrolled in median in the second month after hospital discharge.

**Table 1:** Characteristics of the study population

		DMP only	CR only	Both	None	Total
		N = 83	N = 451	N = 189	N = 371	N = 1094
Age (mean)	Years	70.9	64.9	64.5	69.9	67.0
	(95% CI)	(68.5–73.2)	(63.8–66.0)	(62.9–66.1)	(68.6–71.2)	(66.2–67.7)
Age groups	pct					
25–49		3.6	11.5	12.2	6.2	9.2
50–59		16.9	25.1	22.2	18.9	21.9
60–69		19.3	28.2	27.5	20.5	24.8
70–79		42.2	23.7	33.3	31.0	29.3
80+		18.1	11.5	4.8	23.5	14.9
Male sex	pct	77.1	71.8	68.3	69.5	70.8
	(95% CI)	(66.6–85.6)	(67.4–76.0)	(61.1–74.8)	(64.6–74.2)	(68.1–73.5)
Diabetes	pct	27.7	18.6	9.5	14.8	16.5
	(95% CI)	(18.5–38.6)	(15.1–22.5)	(5.7–14.6)	(11.4–18.9)	(14.3–18.8)
Smoker	pct	19.3	38.1	30.2	28.0	31.9
	(95% CI)	(11.4–29.4)	(33.6–42.8)	(23.7–37.2)	(23.5–32.9)	(29.2–34.8)
Hypertension	pct	91.6	83.2	79.4	83.8	83.4
	(95% CI)	(85.6–97.4)	(79.4–86.5)	(72.9–84.9)	(79.7–87.4)	(81.0–85.5)
Obesity	pct	20.5	25.9	17.5	18.9	21.7
	(95% CI)	(12.4–30.8)	(22.0–30.3)	(12.3–23.6)	(15.0–23.2)	(19.3–24.2)
STEMI	Pct	28.9	51.0	48.7	34.0	43.1
	(95% CI)	(19.5–39.9)	(46.3–55.7)	(41.4–56.0)	(29.2–39.0)	(40.2–46.1)

*DMP* disease management program, *CR* cardiac rehabilitation; smoker and diabetes only relevant if being current at the time of the initial myocardial infarction registered in the data base

age/age groups = age in years at the time of the initial acute myocardial infarction

Those who participated in CR were younger and more often smokers at the time of AMI than those who did not participate (Table 1). In contrast, those who participated in a DMP were less often smokers than those who did not. STEMI was most common among CR participants.

About one third of all participants experienced MACE within 2 years of follow up and 9% experienced a reinfarction (Table 2). Those who participated in DMP had experienced more MACE than those who participated in CR, as evidenced by the deaths in the group of 83 DMP participants that did not take part in CR. The mean age of this subgroup was 71 years and therefore much higher than the average age of all DMP participants.

**Table 2:** Proportion of patients experiencing negative relevant outcomes within 2 years after AMI

	DMP only N = 83	CR only N = 451	Both N = 189	None N = 371	Total N = 1094
MACE1	45.8	14.6	19.1	27.5	22.1
	35.0–57.1	11.5–18.2	13.7–25.4	23.0–32.3	19.7–24.7
MACE2	51.8	24.6	31.2	35.0	31.4
	40.6–62.9	20.7–28.9	24.6–37.8	30.2–40.1	28.6–34.2
Reinfarction	10.8	8.9	7.4	10.2	9.2
	5.1–19.6	6.4–11.9	4.1–12.1	7.4–13.8	7.6–11.1
Stroke	1.2	3.3	2.7	3.0	2.9
	0.0–6.5	1.9–5.4	0.9–6.1	1.5–5.2	2.0–4.1
PCI	9.6	8.7	11.1	10.0	9.6
	4.3–18.1	6.1–11.2	7.0–16.5	7.1–13.5	7.9–11.5
CABG	0.0	5.8	5.8	3.0	4.4
	0.0–4.3	3.8–8.3	2.5–9.2	1.5–5.2	3.3–5.8
Cardiac death	13.3	0.7	2.7	5.7	3.7
	6.8–22.5	0.1–1.9	0.9–6.1	3.5–8.5	2.6–5.0
Death (other)	25.3	2.9	9.0	11.3	8.5
	16.4–36.0	1.5–4.9	5.3–14.0	8.3–15.0	6.9–10.3

MACE 1 = composite endpoint including Reinfarction, Stroke and Death (cardiac / other), MACE 2 = composite endpoint including MACE 1 plus percutaneous coronary intervention (PCI) and coronary artery bypass graft (CABG)

*DMP* disease management program, *CR* cardiac rehabilitation

#### Determinants of DMP enrollment and participation in CR

**Fig. 2:**
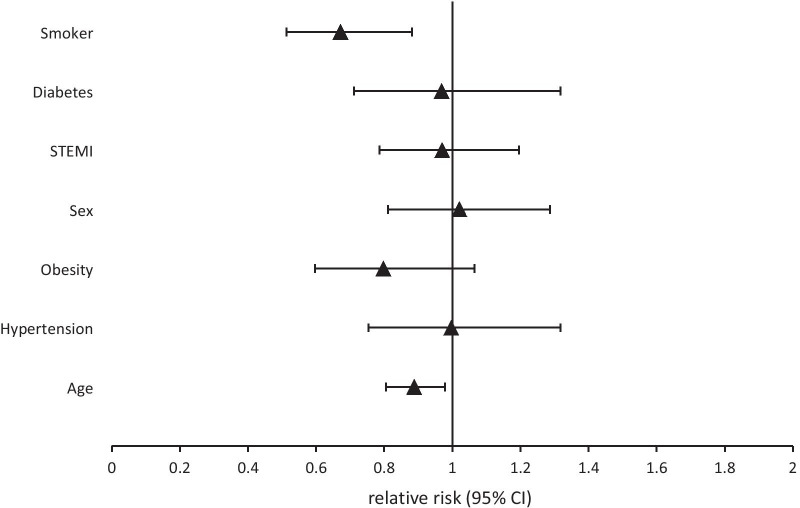
Relative risk for DMP enrollment. Sex: reference = male; STEMI: reference = NSTEMI; age per 10 years; Remaining variables are binary (yes vs. no)

**Fig. 3:**
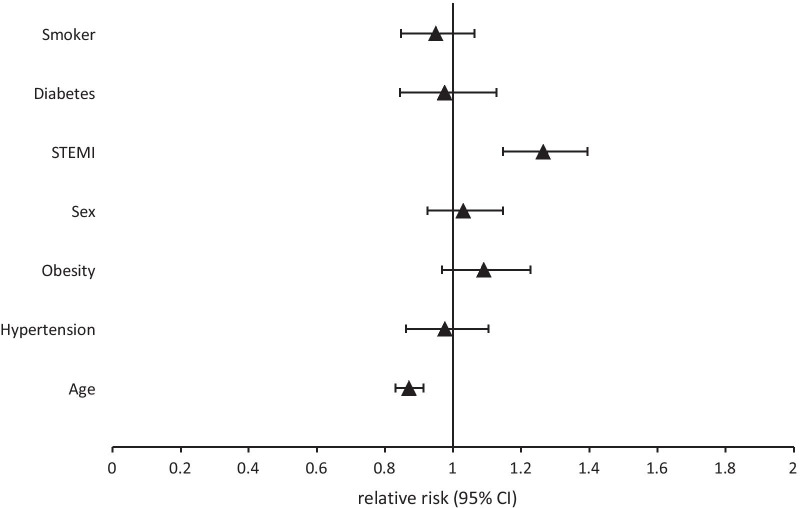
Relative risk for participating in cardiac rehabilitation. Sex reference = male; STEMI = ST-elevation myocardial infarction with reference = non-STEMI; Age = age in years continuously in 10 year steps; Remaining variables are binary (yes vs. no)

In the multivariable model, smoking at the time point of AMI was associated with lower participation in DMP, but not with lower participation in CR (Figs. 2 and 3, Additional file 1: Supplemental Table 1). In contrast, higher age was associated with lower participation in CR but not in DMP. STEMI was also associated with increased participation in CR.

#### Association between participation in DMP or CR after AMI and outcomes during follow up

The comparison of MACE1 and MACE2 showed higher absolute numbers of events and narrower CI for MACE2 without dilution effects. Thus, MACE2 was used as the primary endpoint in all cox regression analyses.

**Fig. 4:**
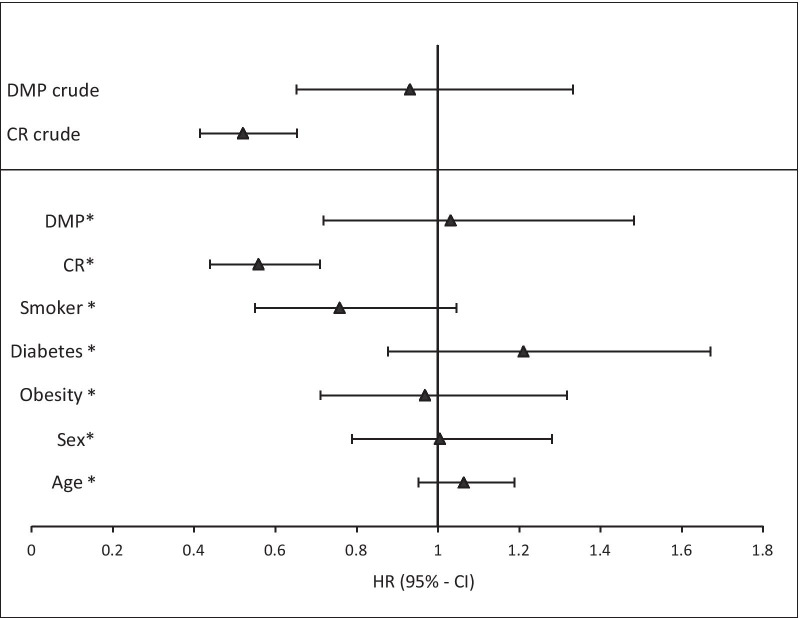
Association between secondary prevention or patient’s risk factors and MACE. Sex: reference = male; STEMI: reference = NSTEMI; Age = age in years continuously. Remaining variables are binary (yes vs. no); * = adjusted model

Participation in DMP was not associated with improved outcomes (crude hazard ratio = 0.93; 95% CI 0.65–1.33), while participation in CR was associated with risk reduction of about 50% (0.52; 0.41–0.65). These results were virtually unchanged after adjustment for age, sex, several diseases and a mutual adjustment for DMP and cardiac rehabilitation (Fig. 4).

Overall, the effects of stratification for the considered subgroups were small indicating that selection of participants according to these variables did not strongly affect the impact of either CR or DMP.

Age, sex and obesity did not show an association with change in survival time in our 2-year observation. Smokers showed a lower hazard rate with the confidence interval still containing the null effect (HR = 0.76; 95% CI 0.55–1.05), similarly there was a slightly increased risk of MACE in participants with diabetes, but the confidence interval included 1 (HR = 1.21; 95% CI 0.88–1.67).

Sensitivity analysis with 77.6% of all DMP participants who began their program after the AMI did not change the outcome noticeably. The HRs for DMP and CR were 0.98 (0.62–1.57) and 0.55 (0.43–0.71), respectively.

When stratified by the time point of smoking cessation, the effect was somewhat stronger in those CR-participants, who stopped smoking before the CR, when compared to those who did not stop smoking before CR or did not smoke (Table 3). However, a strong association between smoking status and CR effect was not found.

**Table 3:** Effects of participation in cardiac rehabilitation on occurrence of MACE1 stratified by smoking status

Model Effects of rehabilitation in those who	Hazard ratio^a^ (95% CI)
…did not smoke at time point of AMI	0.59 (0.45–0.78)
… smoked at time point of AMI	0.45 (0.27–0.74)
… smoked at time point of AMI, but stopped before rehabilitation	0.39 (0.18–0.83)
… smoked at time point of AMI, but did not stop smoking before rehabilitation	0.51 (0.26–1.01)

^a^Participation in cardiac rehabilitation vs. no participation regarding MACE1 occurrence

## Discussion

Using data from the regional myocardial infarction registry RHESA, there was no evidence that participating in DMPs does result in lower rates of cardiac events. On the other hand, participation in CR after discharge from the hospital was associated with a distinctly lower hazard rate of MACE compared to non-participants.

The potential explanation for the lack of specific effect of DMPs could be that patients participating in DMPs did not receive similar care. It could be either that DMPs are not fully implemented, or that patients outside of DMPs benefit from the fact that their GPs employ the rules of DMPs also to them. DMPs have been repeatedly adapted since their establishment in 2003, so there may be a spillover effect on the outpatient treatment by GPs on all patients regardless of being actively enrolled in a DMP or not [17, 20].

We expected to see a higher prevalence of diabetes mellitus, obesity and smoking in DMP participants, due to DMPs targeting the reduction of those risk factors for cardiac events [15]. In contrast to our expectations, in our cohort patients with risk factors like smokers and obese people were found to have a lower likelihood to be enrolled in DMPs. On this account, several key components of the DMPs probably could not achieve their full effect, because patients who would likely benefit the most were participating less often in DMPs. The slightly lower hazard ratio for MACE in the crude model is probably due to the DMP participants being already healthier before the DMP than the control group.

It is also remarkable, that the proportion of enrollment in DMPs of about one quarter is substantially lower than the 77% participantion rate found 8 years ago in a comparable study in the region of Augsburg by Laxy et al. [16]. Röttger et al. [23] found similar results (enrollment rate of 72%) throughout Germany in 2013 in patients with CHD. Possible explanations could be regional socio-economic differences [43] and health characteristics of the respective cohorts as well as the time span between the studies [21, 23]. In conclusion, it is apparent that the DMPs are currently ineffective in reaching their required target group in Saxony-Anhalt. While only about one third of all RHESA registered patients took part in the baseline survey with 70% answering in the respective follow-up, often those who participated were more health conscious.

Thus, our results indicate that the process of DMP participant acquisition, which does not reach the high-risk population, may be one of the reasons for the lack of effects on MACE in patients after myocardial infarction. This is especially important, considering the higher rates of cardiac mortality, risk factor distribution and demographic structure in our regional study population [37]. These observations are in line with a study by Schäfer et al. about selection effects in current DMP research [35]. While our results match the conclusions of similar studies [16, 17], health insurance evaluations repeatedly described protective effects [36]. The explanation could be a different comparison group.

In contrast, participation in CR after discharge from hospital resulted in a distinctly lower hazard rate of MACE compared to non-participants. There is a possibility that this effect may be related to self-selection of the participants of CR. On the one hand, we found that many of those who smoked at the time of AMI, stopped smoking before starting CR. This could indicate that there is an underlying motivation for lifestyle changes resulting in the participation in CR. Such motivation rather than the CR itself could be responsible for the positive effect attributed to CR. Consistently, there was an indication of more beneficial effects of CR in this subgroup. On the other hand, adjusting for the relevant risk factors did not pertinently change the estimated effect of CR in the direction of the null. We conclude that selection is likely present in CR participation and enhancing the observed effect, but it does not explain it fully.

According to our results, smoking at the time of the initial AMI shows a weak association with prolonged survival time. This well-known ‘smoker’s paradox’ has been reported in several studies [38, 39]. The main suspected reason is that smokers experience AMI at younger age, and thus the relation with mortality is diluted.

### Strengths and limitations

The strength of this study lies in the prospective, population-based design with a cohort of patients severely affected by the elevated risk of multiple complications after their myocardial infarction. In addition, the time-dependent covariate design in survival time analysis as well as the possibility to adjust the regression to multiple important co-morbidities and patient’s characteristics with relevant influence on the total effect adds to the novelty and importance in current research.

Immortal time bias is common in prospective cohort studies, but our method of implementing DMP status as a time dependent covariate in the analysis can strongly reduce biased treatment effect estimates [32, 42]. This enables our analysis to account for DMP time whether or not the patient enrolled before or after the time of the initial AMI. Hence, our study is not limited by a time-fixed control group status which would ignore late onset DMP enrollment.

The findings of our study are limited by the follow-up time of only 2 years, and thus later outcomes are not considered. A longer follow-up period with greater, Germany-wide data could add to our results. Also, the main source of our data are self-reported questionnaires and telephone interviews which allow for recall bias or erroneous answers [40]. In order to investigate if the observed effects of either DMP or CR were influenced by selection of participants in those programs, we conducted multivariable analysis [21, 41]. Although we selected the covariates for the analysis by directed acyclic graphs, this choice was also limited by the availability of data. Adjusting our analysis for socioeconomic status was not possible, because of 118 patients who died and did not previously provide this information. Various mechanisms could affect the representativeness of the sample of AMI survivors in our study. First, the proportion of those included in RHESA in comparison to all AMIs in the study regions was at maximum 85% in 2016 and substantially lower in subsequent years. Second, only patients who agreed to the follow up were included (38% of patients who were alive at discharge). We do not have information to what degree this participation was a random process. The patients included in RHESA who agreed to follow up were on average about 4 years younger than those for whom agreement to follow up was not available, but for most other characteristics we saw no difference between participants and non-participants (Additional file 1: Supplental Table 2).

## Conclusions

Within the framework of the regional AMI registry in urban and rural regions of the federal state (RHESA), we could not confirm a benefit from participating in DMP for AMI survivors with respect to MACE. The present findings suggest that the reason for the lack of effects of DMPs may be the insufficient inclusion of those who would benefit from the DMP. In contrast, we observed a positive effect of CR, although it is possible that these results are confounded due to differences in personal motivation to participation. Thus, selection effects should be considered.

## Supplementary Information

**Additional file 1.** **Table 1**. Relative Risk for participation in DMP or CR with corresponding 95% Confidence Intervals. **Table 2.** Characteristic of participants and non-participants of RHESA follow up.

## Data Availability

The datasets used and/or analyzed during the current study are available from the corresponding author on reasonable request.

## Abbreviations

AMI: Acute myocardial infarction
CABG: Coronary artery bypass graft
CHD: Coronary heart disease
CI: Confidence interval
CR: Cardiac rehabilitation
GP: General practitioner
MACE: Major adverse cardiac events
NSTEMI: Non-ST-elevation myocardial infarction
PCI: Percutaneous coronary intervention
RC1: RHESA-CARE 1
RC3: RHESA-CARE 3
RHESA: Regional Myocardial Infarction Registry
STEMI: ST-elevation myocardial infarction
