# Supplementary material for: Participation in disease management programs and major adverse cardiac events in patients after acute myocardial infarction: a longitudinal study based on registry data
Source: BMC Cardiovasc Disord. 2021 Jan 6;21:18. doi: 10.1186/s12872-020-01832-3 (PMC7788767; doi:10.1186/s12872-020-01832-3)
Supplement: Supplementary file 1 — Additional file 1. Table 1. Relative Risk for participation in DMP or CR with corresponding 95% Confidence Intervals. Table 2. Characteristic of participants and non-participants of RHESA follow up. [file 12872_2020_1832_MOESM1_ESM.docx]

**Supplemental material**

**Supplemental Table 1:** Relative Risk for participation in DMP or CR with corresponding 95% Confidence Intervals

| **variable** | **Relative Risk** | **Lower 95% CI-limit** | **Higher 95% CI-limit** |
| --- | --- | --- | --- |
|  | **DMP participation** | | |
| **Smoker** | 0.67 | 0.51 | 0.88 |
| **Diabetes** | 0.97 | 0.71 | 1.32 |
| **STEMI** | 0.97 | 0.79 | 1.20 |
| **Sex (ref. = male)** | 1.02 | 0.81 | 1.29 |
| **Obesity** | 0.80 | 0.60 | 1.07 |
| **Hypertension** | 1.00 | 0.75 | 1.32 |
| **Age** | 0.89 | 0.81 | 0.98 |
|  | **CR participation** | | |
| **Smoker** | 0.95 | 0.85 | 1.06 |
| **Diabetes** | 0.98 | 0.84 | 1.13 |
| **STEMI** | 1.26 | 1.15 | 1.40 |
| **Sex (ref. = male)** | 1.03 | 0.93 | 1.15 |
| **Obesity** | 1.09 | 0.97 | 1.23 |
| **Hypertension** | 0.98 | 0.86 | 1.10 |
| **Age** | 0.87 | 0.83 | 0.91 |
| DMP = Disease Management Program;  CR = cardiac rehabilitation,  STEMI = ST-elevation myocardial infarction | | | |

**Supplemental Table 2:** Characteristic of participants and non-participants of RHESA follow up

|  |  | **Participants** | **Non-Participants** |
| --- | --- | --- | --- |
| Age [years] | Mean ±SD | 67.1 ±12.8 | 71.4 ±13.3 |
| Male sex | n (%) | 1,213 (68 %) | 1,846 (64 %) |
| Hypertension | n (%) | 1,432 (82 %) | 2,311 (85 %) |
| Overweight | n (%) | 847 (75 %) | 1,196 (71 %) |
| Diabetes mellitus | n (%) | 584 (34 %) | 1,008 (37 %) |
| Smoker | n (%) | 545 (37 %) | 610 (31 %) |
| RHESA = regional register of myocardial infarction in Saxony-Anhalt | | | |
